# PW03-014 - TLR4 and MEFV variants are Behçet's risk factors

**DOI:** 10.1186/1546-0096-11-S1-A240

**Published:** 2013-11-08

**Authors:** Y Kirino, Q Zhou, Y Ishigatsubo, N Mizuki, Y Kim, DL Kastner, A Gül, EF Remmers

**Affiliations:** 1 Department of Internal Medicine and Clinical Immunology, Yokohama City University Graduate School of Medicine, Yokohama, Japan; 2 Inflammatory Disease Section, Medical Genetics Branch, NIH/NHGRI, Bethesda, USA; 3 Department of Ophthalmology and Visual Science, Yokohama City University Graduate School of Medicine, Yokohama, Japan; 4 Genometrics Section, Inherited Disease Research Branch, NIH/NHGRI, Baltimore, USA; 5 Istanbul Faculty of Medicine, Department of Internal Medicine, Division of Rheumatology, Istanbul University, Istanbul, Turkey

## Introduction

Genome-wide association studies (GWAS) are a powerful means for identifying genes with disease-associated common variants, but they are not well-suited to detect genes with disease-associated rare or low-frequency variants. It has long been debated whether the innate immune system is involved in the pathogenesis of Behçet's disease (BD) but genetic evidence to support this hypothesis is sparse.

## Objectives

To determine whether rare and low frequency variants in genes involved in innate immunity are associated with BD.

## Methods

In the current study, non-synonymous variants (NSVs) identified by deep exonic resequencing of 10 genes found by GWAS (*IL10*, *IL23R*, *CCR1*, *STAT4*, *KLRK1*, *KLRC1*, *KLRC2*, *KLRC3*, *KLRC4*, and *ERAP1*) and 11 genes selected for their role in innate immunity (*IL1B*, *IL1R1*, *IL1RN*, *NLRP3*, *MEFV*, *TNFRSF1A*, *PSTPIP1*, *CASP1*, *PYCARD*, *NOD2*, and *TLR4*) were evaluated for BD association in Japanese and Turkish populations. A differential distribution of the rare and low frequency NSVs of each gene in 2461 BD cases compared with 2458 controls was evaluated by three different burden tests.

## Results

By stringent criteria requiring at least one burden test with study-wide significance (p < 0.0024) and a corroborating test with at least nominal significance (p < 0.05), rare and low frequency NSVs in one GWAS-identified gene, *IL23R* (p = 6.9 x 10^-5^), and one gene involved in innate immunity, *TLR4* (p = 8.0 x 10^-4^), were associated with BD. In addition, damaging or rare damaging *NOD2* variants were nominally significant across all three burden tests applied (p = 0.0063 to 0.045). Furthermore, carriage of *MEFV*-M694V, but not other *MEFV* mutations known to cause recessively inherited familial Mediterranean fever, conferred BD risk in the Turkish population (OR = 2.65, p = 1.8 x 10^-12^).

## Conclusion

Rare and low frequency NSVs of two novel BD-associated genes, *MEFV* and *TLR4*, implicate innate immune and bacterial sensing mechanisms in BD pathogenesis. Furthermore, disease-associated *IL23R* rare and low frequency NSVs add to the common variant GWAS evidence implicating this locus.

## Disclosure of interest

None declared

